# Supplementary material for: Adherence to a Healthy Diet and Risk of Multiple Carotid Atherosclerosis Subtypes: Insights from the China MJ Health Check-Up Cohort
Source: Nutrients. 2024 Jul 19;16(14):2338. doi: 10.3390/nu16142338 (PMC11280435; doi:10.3390/nu16142338)
Supplement: Supplementary file 1 [file nutrients-16-02338-s001.zip › nutrients-3095619-supplementary.pdf]

Supplementary material

Adherence to a healthy diet and risk of multiple carotid atherosclerosis subtypes: insights from the China MJ health check-up cohort

Figure S1. Flow chart of the analyses

Figure S2. Directed acyclic graph (DAG) derived from previous literature and expert knowledge

Figure S3. The restricted cubic spline for the association between various dietary pattern scores and CP in the whole population

Figure S4. Stratified analysis of estimated associations between various dietary patterns and CP according to sex and lifestyles, by comparing the highest with the lowest quartiles

Figure S5. The potential synergistic effect between lifestyles and DDS on risk of CAS

Figure S6. Associations between dietary pattern scores and metabolic syndrome components levels

Supplementary Materials Section S1. The calculation of a priori dietary patterns

Supplementary Materials Section S2. The calculation of a posteriori dietary patterns

Supplementary Materials Section S3. The definition of low-risk lifestyles

Supplementary Materials Section S4. Measures and definitions of covariates

Table S1. Association of the dietary pattern scores with CAS in the sensitivity analysis 1

Table S2. Association of the dietary pattern scores with CAS in the sensitivity analysis 2

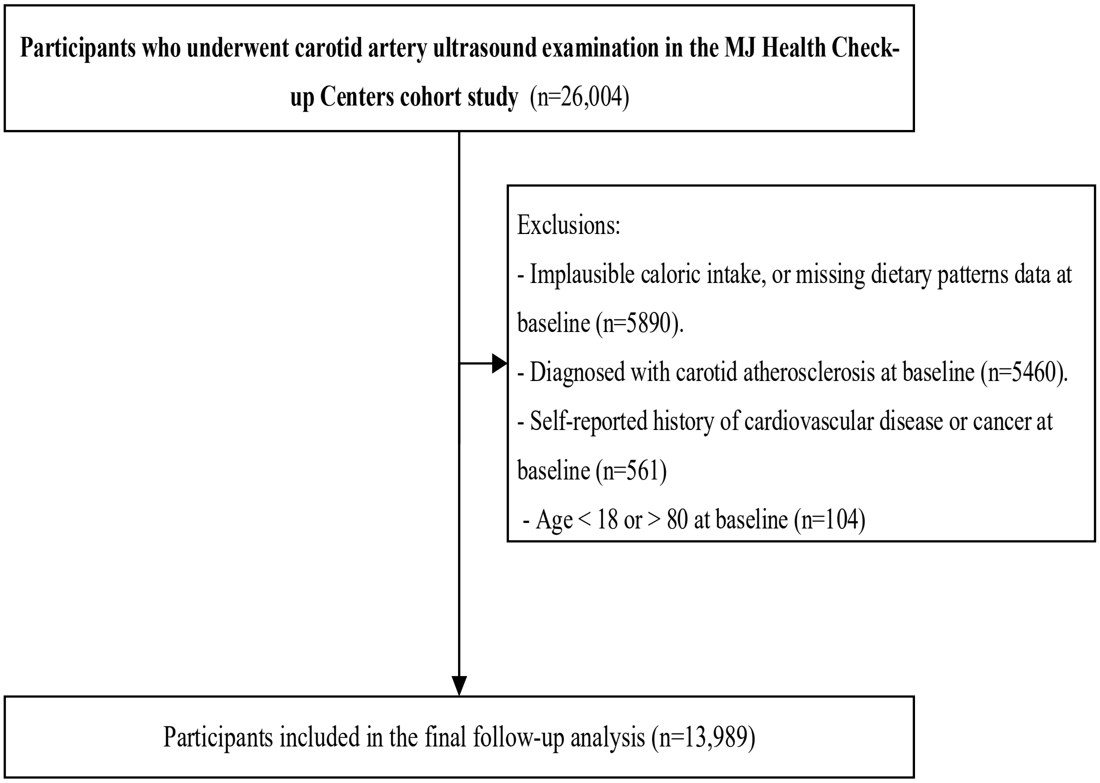

Figure S1. Flow chart of the analyses

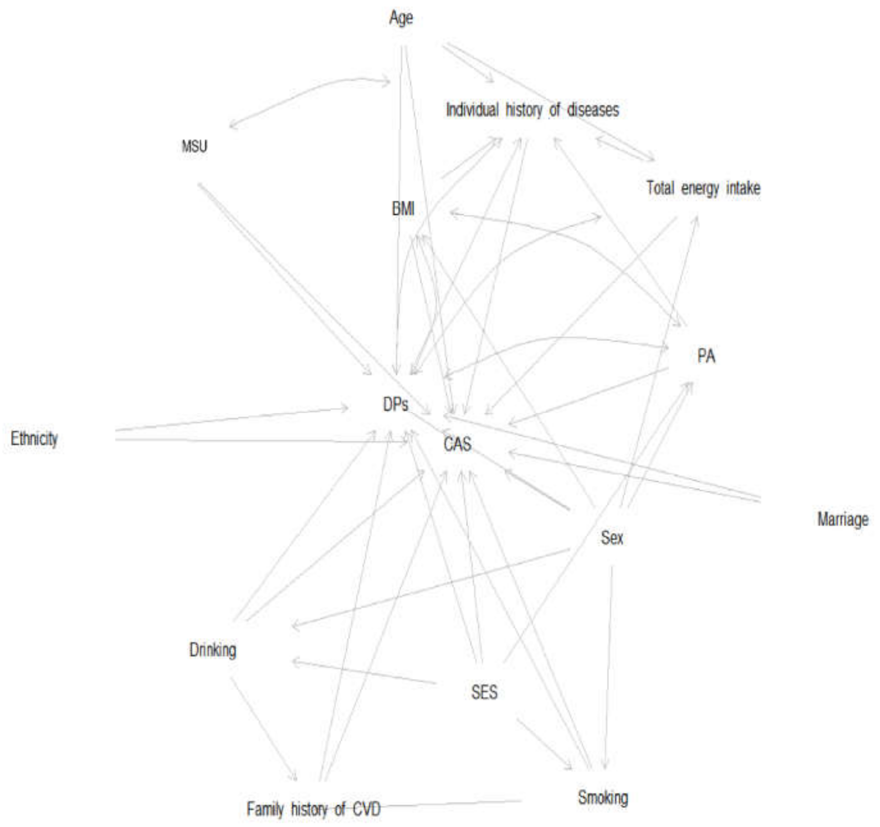

Figure S2. Directed acyclic graph (DAG) derived from previous literature and expert knowledge. Arrows represent causal associations. DPs are exposure, and CAS is the outcome. Other variables represent confounding factors. SES: socioeconomic status (including educational attainment and annual income); BMI: body mass index; CAS: carotid atherosclerosis; CVD: cardiovascular disease; DPs: dietary patterns; PA: physical activity; MSU: multivitamin supplement use; Individual history of disease (including hypertension, diabetes, and dyslipidemia).

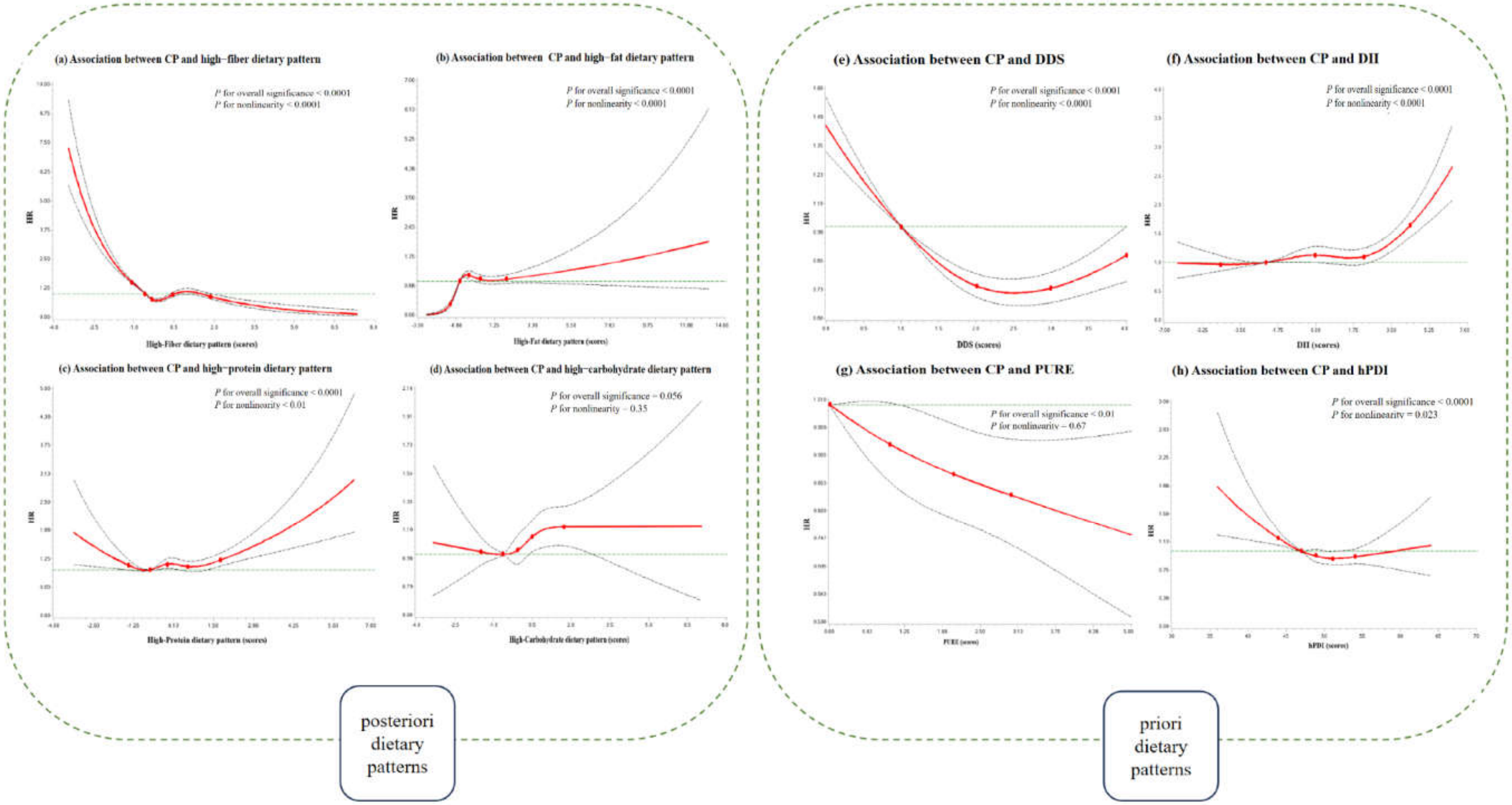
(a) Association between CP and high−fiber dietary pattern
P for overall significance < 0.0001
P for nonlinearity < 0.0001
HR
High-Fiber dietary pattern (scores)
(b) Association between CP and high−fat dietary pattern
P for overall significance < 0.0001
P for nonlinearity < 0.0001
HR
High-Fat dietary pattern (scores)
(c) Association between CP and high−protein dietary pattern
P for overall significance < 0.0001
P for nonlinearity < 0.01
HR
High-Protein dietary pattern (scores)
(d) Association between CP and high−carbohydrate dietary pattern
P for overall significance = 0.056
P for nonlinearity = 0.35
HR
High-Carbohydrate dietary pattern (scores)
(e) Association between CP and DDS
P for overall significance < 0.0001
P for nonlinearity < 0.0001
HR
DDS (scores)
(f) Association between CP and DII
P for overall significance < 0.0001
P for nonlinearity < 0.0001
HR
DII (scores)
(g) Association between CP and PURE
P for overall significance < 0.01
P for nonlinearity = 0.67
HR
PURE (scores)
(h) Association between CP and hPDI
P for overall significance < 0.0001
P for nonlinearity = 0.023
HR
hPDI (scores)
posteriori dietary patterns
priori dietary patterns

Figure S3. The restricted cubic spline for the association between various dietary pattern scores and CP in the whole population. Solid red lines and dashed black lines represent HR and 95% CI based on restricted cubic splines in the Cox regression model. The horizontal green dashed line represents the reference value. Knots were placed at the 5th, 25th, 50th, 75th and 95th percentiles of the dietary patterns scores distribution, and the reference value was set at the 25th percentile. Adjustment factors were age, sex, ethnicity, educational attainment, marital status, household income, physical activity, smoking status, drinking status, multivitamin supplement use, BMI, family history of cardiovascular disease, individual history of diabetes, hypertension, and dyslipidemia, total energy intake, and each other dietary pattern scores (i.e., High-Fiber, High-Fat, High-Protein, and High-Carbohydrate) for posteriori dietary patterns. Adjustment factors were age, sex, ethnicity, educational attainment, marital status, household income, physical activity, smoking status, drinking status, multivitamin supplement use, BMI, family history of cardiovascular disease, individual history of diabetes, hypertension, and dyslipidemia and total energy intake for priori dietary patterns. BMI: body mass index; CI: confidence interval; CP: carotid plaque; DDS dietary diversity score; DII: dietary inflammation index; hPDI: healthful plant-based diet index; HR: hazard ratio; PURE: Prospective Urban Rural Epidemiology healthy diet.

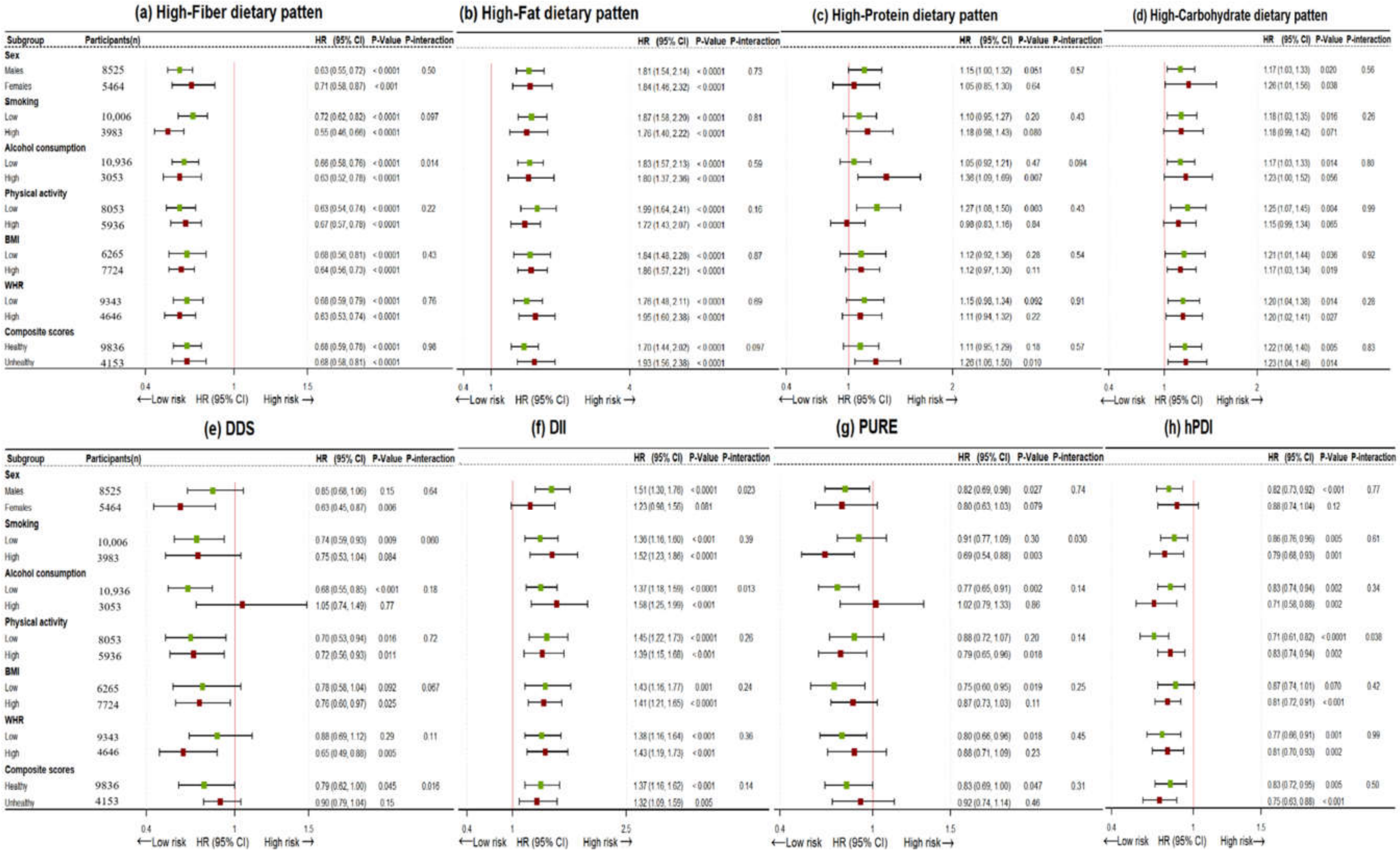

(a) High-Fiber dietary patten
(b) High-Fat dietary patten
(c) High-Protein dietary patten
(d) High-Carbohydrate dietary patten
(e) DDS
(f) DII
(g) PURE
(h) hPDI
Subgroup
Participants(n)
HR (95% CI)
P-Value
P-interaction
Sex
Males 8525
Females 5464
Smoking
Low 10,006
High 3983
Alcohol consumption
Low 10,936
High 3053
Physical activity
Low 8053
High 5936
BMI
Low 6265
High 7724
WHR
Low 9343
High 4646
Composite scores
Healthy 9836
Unhealthy 4153
←Low risk HR (95% CI) High risk→

Figure S4. Stratified analysis of estimated associations between various dietary patterns and CP according to sex and lifestyles, by comparing the highest with the lowest quartiles. Analyses of priori dietary patterns were adjusted for age, sex, ethnicity, educational attainment, marital status, household income, physical activity, smoking status, drinking status, multivitamin supplement use, BMI, family history of cardiovascular disease, individual history of diabetes, hypertension, and dyslipidemia and total energy intake, with exclusion of the stratified variables as appropriate. Additionally adjusted each other dietary pattern scores (i.e., High-Fiber, High-Fat, High-Protein, and High-Carbohydrate) for posteriori dietary patterns. *P* for overall interaction was calculated using likelihood ratio test. BMI: body mass index; CI: confidence interval; CP: carotid plaque; DDS dietary diversity score; DII: dietary inflammation index; hPDI: healthful plant-based diet index; HR: hazard ratio; PURE: Prospective Urban Rural Epidemiology healthy diet; WHR: waist-to-hip ratio.

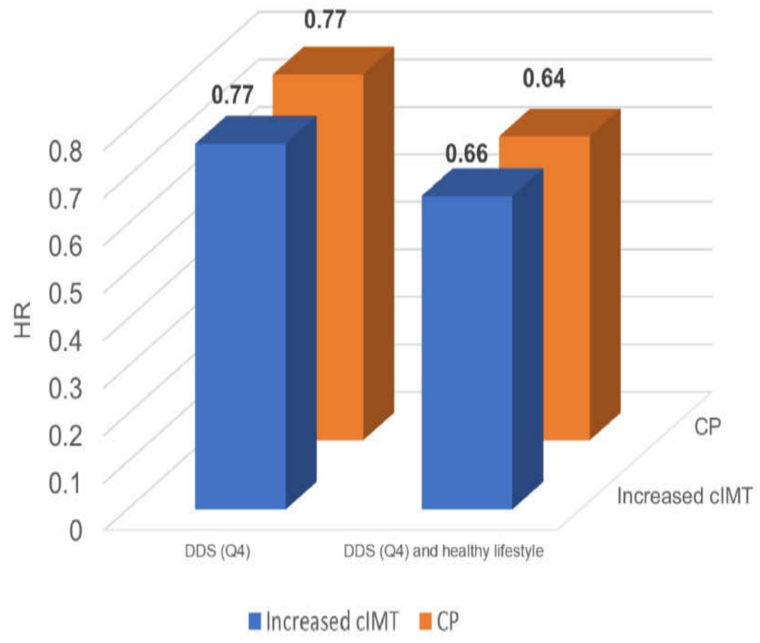

Figure S5. The potential synergistic effect between lifestyles and DDS on risk of CAS. We analyze hazard ratios (HRs) and 95% confidence intervals (CI) for the joint effect of lifestyles and DDS on CAS risk based on the results of above interaction effects. In the separate and joint analyses, we used a grouping approach that combined DDS with a healthy or unhealthy lifestyle (e.g., category 1: DDS[Q1]/unhealthy lifestyle +; category 3: DDS[Q4]/ healthy lifestyle-; category 2: other combinations), which DDS/lifestyle status were divided into three groups. The figure displays the results comparing the lowest risk group to the highest risk group. Adjustment factors were age, sex, ethnicity, educational attainment, marital status, household income, physical activity, smoking status, drinking status, multivitamin supplement use, BMI, family history of cardiovascular disease, individual history of diabetes, hypertension, and dyslipidemia and total energy intake, with exclusion of the joint effect variables as appropriate. BMI: body mass index; CI: confidence interval; CP: carotid plaque; DDS dietary diversity score; HR: hazard ratio.

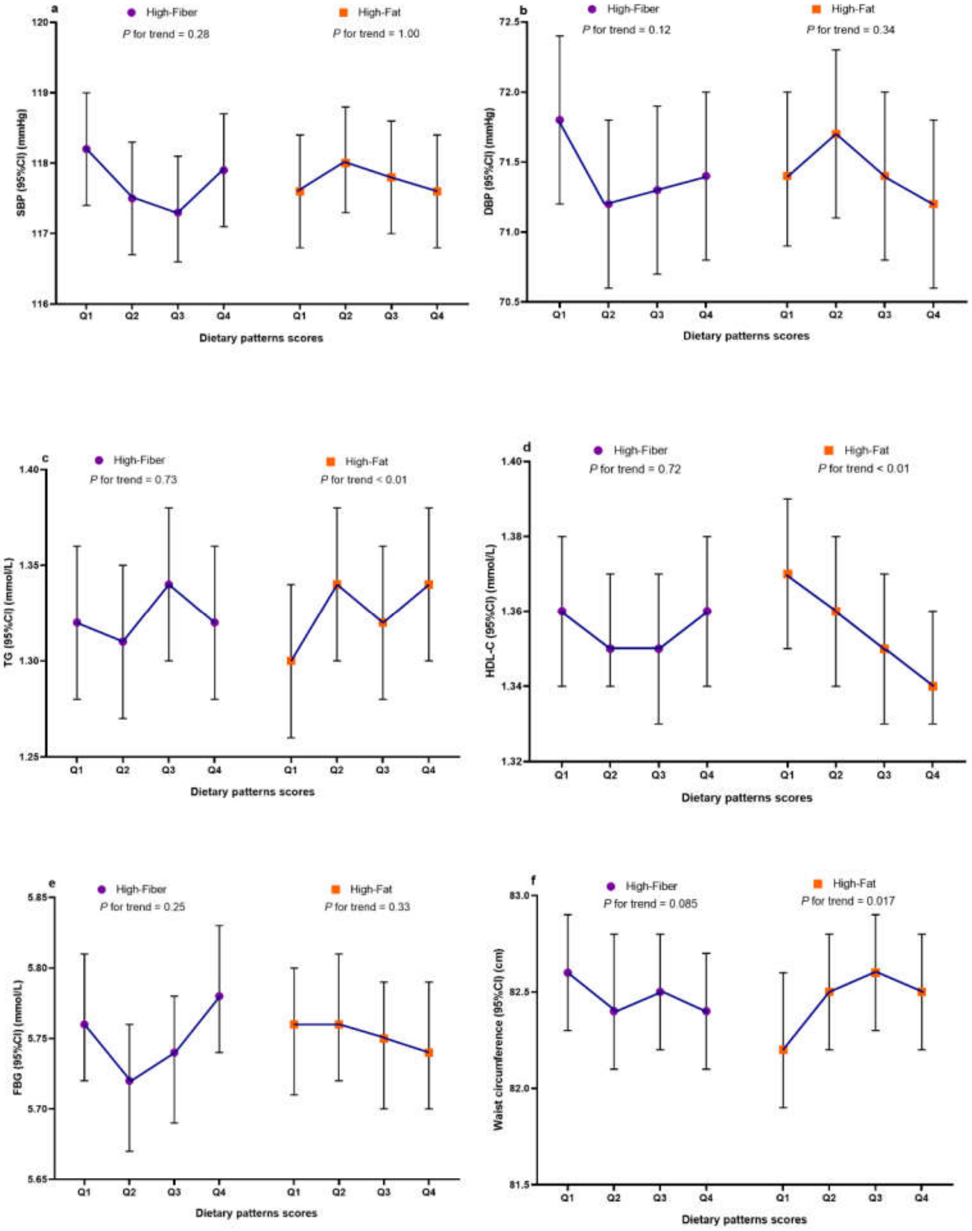

Figure S6. Associations between dietary pattern scores and metabolic syndrome components levels. Generalized linear models were used to analyze differences and trends in metabolic syndrome component levels (the average from the second record to the last record) between dietary pattern score quartiles (baseline). Multivariable models were adjusted for age, sex, ethnicity, educational attainment, marital status, household income, physical activity, smoking status, drinking status, multivitamin supplement use, BMI, family history of cardiovascular disease. BMI; body mass index; TG: triglycerides; HDL-C: high-density lipoprotein cholesterol; SBP: systolic blood pressure; DBP: diastolic blood pressure; FBG: fasting blood glucose.

Supplementary Materials Section S1. The calculation of a priori dietary patterns

To represent adherence to a dietary diversity, the food items were aggregated into 7 major food group based on a balanced diet pagoda: vegetables, fruits, eggs, dairy and dairy products, legumes and legume products, livestock meat (including pork, poultry, beef, and organs), and fish and shrimp (including seafood, freshwater fish, and aquatic products). We did not include grain or oil in the construction of the DDS because almost all Chinese individuals consumed these 2 foods every day, similar to previous studies [1]. Compared to previous studies that calculated DDS [2,3], we excluded the food group component of tea and gastric due to the lack of a separate food group for tea and garlic consumption. The frequency response options for these food item questions were categorized as "≥ 1 serving/day" as "often or almost every day", "never or < 1 serving per week" as "rarely or never", and the remaining frequency options as "occasionally". “Often or almost every day” consumption of any food group without considering a minimum intake was categorized as one DDS unit; 7 points of DDS represented the highest level of dietary diversity [2]. The total score is categorized as scores 0-1, 2, 3, ≥ 4. To assess the inflammatory capacity of the diet, the DII was calculated with dietary data derived from a 25-item FFQ and described in detailed elsewhere [4]. Briefly, the dietary data for each study participant was first linked to the regionally representative global database of dietary survey from 11 countries for each of the 45 parameters (i.e., foods, nutrients, and other food constituents). This global database provides a robust estimate of a mean and standard deviation for each of the food parameters considered. A z-score was derived by subtracting the “standard global mean” from the amount reported and then dividing this value by the standard deviation. This value was then converted to a centered percentile score, which was then multiplied by the respective food parameter inflammatory effect score to obtain the subject’s food parameter-specific DII score. All of the food parameter-specific DII scores were then summed to create the overall DII score for each participant. In the current study, DII scores was calculated based on 29 available food parameters, which were as follows: energy, carbohydrate, protein, total fat, saturated fatty acids, mono-unsaturated fatty acids, polyunsaturated fatty acids, fiber, cholesterol, niacin, thiamine, riboflavin, folate, vitamin A, β-Carotene, vitamin C, vitamin D, vitamin E, vitamin B12, vitamin B6, iron, magnesium, selenium, zinc, omega 6, omega 3, isoflavones, alcohol, and anthocyanidins. To derive PURE, we used an unweighted score based on five food categories each of which have been associated with a lower risk of mortality in the PURE cohort [5]. We eliminated the food group for nuts due to the lack of a separate food group for nut consumption. Lastly, these food categories consisted of fruit,

vegetables, legumes, fish, and dairy. A value of 0 or 1 was assigned to each of the five components of the score with the use of the median in the study cohort as the cut-off. A score of 1 (healthy) was assigned when an individual's intake of the food component was above the median in this population. A score of 0 (unhealthy) was assigned when intake was at or below the median. The total PURE healthy diet score was the sum of the five component scores. The healthy diet scores range from 0 to 5 points, with higher scores indicating a healthier diet. The total score is categorized as scores 0, 1, 2, ≥3. To calculate hPDI, we assigned scores for each food group according to the intake frequency: a score of 5 was assigned for the highest frequency and 1 for the lowest frequency regarding the intake of healthy plant food groups (whole grains, fruits, vegetables, tubers, legumes) (positive scores); a score of 1 was assigned for the highest frequency and 5 for the lowest frequency regarding the intake of less healthy plant (sugar, refined grains, and salt-preserved vegetable) and animal food groups [6,7]. Therefore, the higher the frequency of eating healthy plant foods and the lower the frequency of eating less healthy plant and animal food groups, the higher the hPDI. Except for the DDS and PURE score, the dietary pattern scores were categorized into quartiles for the entire study population.

Reference:

1. Liu, D.; Zhang, X.R.; Li, Z.H.; Zhang, Y.J.; Lv, Y.B.; Wang, Z.H.; Shen, D.; Chen, P.L.; Zhong, W.F.; Huang, Q.M., et al. Association of dietary diversity changes and mortality among older people: A prospective cohort study. *Clin Nutr* **2021**, *40*, 2620-2629, doi:10.1016/j.clnu.2021.04.012.
2. Zheng, J.; Zhou, R.; Li, F.; Chen, L.; Wu, K.; Huang, J.; Liu, H.; Huang, Z.; Xu, L.; Yuan, Z., et al. Association between dietary diversity and cognitive impairment among the oldest-old: Findings from a nationwide cohort study. *Clin Nutr* **2021**, *40*, 1452-1462, doi:10.1016/j.clnu.2021.02.041.
3. Wang, X.M.; Zhong, W.F.; Li, Z.H.; Chen, P.L.; Zhang, Y.J.; Ren, J.J.; Liu, D.; Shen, Q.Q.; Yang, P.; Song, W.Q., et al. Dietary diversity and frailty among older Chinese people: evidence from the Chinese Longitudinal Healthy Longevity Study. *Am J Clin Nutr* **2023**, *117*, 383-391, doi:10.1016/j.ajcnut.2022.11.017.
4. Shivappa, N.; Steck, S.E.; Hurley, T.G.; Hussey, J.R.; Hebert, J.R. Designing and developing a literature-derived, population-based dietary inflammatory index. *Public health nutrition* **2014**, *17*, 1689-1696, doi:10.1017/S1368980013002115.
5. Mente, A.; Dehghan, M.; Rangarajan, S.; O'Donnell, M.; Hu, W.; Dagenais, G.; Wielgosz, A.; S, A.L.; Wei, L.; Diaz, R., et al. Diet, cardiovascular disease, and mortality in 80 countries. *Eur Heart J* **2023**, *44*, 2560-2579, doi:10.1093/eurheartj/ehad269.
6. Chen, H.; Shen, J.; Xuan, J.; Zhu, A.; Ji, J.S.; Liu, X.; Cao, Y.; Zong, G.; Zeng, Y.; Wang, X., et al. Plant-based dietary patterns in relation to mortality among older adults in China. *Nat Aging* **2022**, *2*, 224-230, doi:10.1038/s43587-022-00180-5.
7. Wang, S.; Li, W.; Li, S.; Tu, H.; Jia, J.; Zhao, W.; Xu, A.; Xu, W.; Tsai, M.K.; Chu, D.T., et al. Association between plant-based dietary pattern and biological aging trajectory in a large prospective cohort. *BMC Med* **2023**, *21*, 310, doi:10.1186/s12916-023-02974-9.

Supplementary Materials Section S2. The calculation of a posteriori dietary patterns

For this study, single food items from the FFQ were aggregated into 16 predefined food groups according to their nutritional composition based on the latest available Chinese Food Composition Table **(Table 2)** [8]. The factorability of the correlation matrix of the food groups were preliminarily assessed using Bartlett's test of sphericity (P < 0·0001) and the Kaiser-Meyer-Olkin (KMO) measures of sampling adequacy (KMO statistic = 0·67). Given the reassuring results, an exploratory factor analysis was used to derive dietary patterns based on the above-mentioned 16 food groups. The number of factors to retain were chosen based on the following criteria: factor eigenvalues > 1.0; scree plot construction; and factor interpretability [9]. After a varimax rotation to the factor loading matrix, food groups were applied with a factor loading ≥ |0·30| as the main contributors to dietary pattern and representative of the character of each factor. The 'dominant dietary patterns' were used to label each factor pattern as high-fiber, high-protein, high-fat, and high-carbohydrate dietary pattern, which explained approximately 41·8% of the variance of the food groups.

Reference:

8. Yang, Y.; Wang, G.; Pan, X. China food composition. 2nd ed. *Beijing: Peking University Medical Press* **2009**.
9. Johnson, R.A.; Wichern, D.W. Applied Multivariate Statistical Analysis, 5th ed. *Prentice Hall: Upper Saddler River, NJ, USA.* **2002**.

Supplementary Materials Section S3. The definition of low-risk lifestyles

For smoking, the low-risk group was defined as nonsmokers due to most former smokers quit because of illness [10]. For alcohol consumption, the low-risk group was defined as those who drank less than 15 g alcohol per day [11]. For physical activity, the low-risk group was defined as those who engaged in a sex-specific median or higher level of physical activity [12]. For general adiposity measured by BMI, the low-risk group was defined to those who had a BMI of 18·5-23·9 kg/m^2, the standard classification of normal weight specific for Chinese [13]. For central adiposity measured by WHR, the low-risk group was defined as those who had a WHR<0·90 in males and <0·85 in females [14]. Adiposity measures were used to assess energy balance, a critical aspect of cardiovascular-healthy diet [15]. We assigned 0 ("low risk") or 1 ("high risk") for each lifestyle factor. The healthy lifestyle score was the sum of the scores of 5 factors, ranging from 0 to 5 points (with higher scores indicating higher adherence to a healthy lifestyle). Lifestyles were categorized as healthy (3-5 healthy lifestyle factors) and unhealthy (0-2 healthy lifestyle factors) based on the distribution of lifestyle scores.

Reference:

10. Chen, Z.; Peto, R.; Zhou, M.; Iona, A.; Smith, M.; Yang, L.; Guo, Y.; Chen, Y.; Bian, Z.; Lancaster, G., et al. Contrasting male and female trends in tobacco-attributed mortality in China: evidence from successive nationwide prospective cohort studies. *Lancet* **2015**, *386*, 1447-1456, doi:10.1016/S0140-6736(15)00340-2.
11. Lourida, I.; Hannon, E.; Littlejohns, T.J.; Langa, K.M.; Hypponen, E.; Kuzma, E.; Llewellyn, D.J. Association of Lifestyle and Genetic Risk With Incidence of Dementia. *JAMA* **2019**, *322*, 430-437, doi:10.1001/jama.2019.9879.
12. Lv, J.; Yu, C.; Guo, Y.; Bian, Z.; Yang, L.; Chen, Y.; Tang, X.; Zhang, W.; Qian, Y.; Huang, Y., et al. Adherence to Healthy Lifestyle and Cardiovascular Diseases in the Chinese Population. *J Am Coll Cardiol* **2017**, *69*, 1116-1125, doi:10.1016/j.jacc.2016.11.076.
13. Chen, C.; Lu, F.C.; Department of Disease Control Ministry of Health, P.R.C. The guidelines for prevention and control of overweight and obesity in Chinese adults. *Biomed Environ Sci* **2004**, *17 Suppl*, 1-36.
14. Organization, W.H. Waist circumference and waist–hip ratio. *Report of a WHO expert consultation, Geneva, 8-11 December 2008. Geneva: World Health Organization; 2011*.
15. Lloyd-Jones, D.M.; Hong, Y.; Labarthe, D.; Mozaffarian, D.; Appel, L.J.; Van Horn, L.; Greenlund, K.; Daniels, S.; Nichol, G.; Tomaselli, G.F., et al. Defining and setting national goals for cardiovascular health promotion and disease reduction: the American Heart Association's strategic Impact Goal through 2020 and beyond. *Circulation* **2010**, *121*, 586-613, doi:10.1161/CIRCULATIONAHA.109.192703.

Supplementary Materials Section S4. Measures and definitions of covariates

BMI is calculated by dividing weight in kilograms by the square of height in meters. WC was measured at the umbilical level with subjects standing and breathing normally. Hypertension was defined as SBP/DBP ≥ 140/90 mmHg, confirmed hypertension medication use, or self-reported history of hypertension. All participants provided blood samples following an overnight fast of at least 8 hours. The samples were analyzed using standardized devices and procedures to measure fasting blood glucose (FBG), total cholesterol (TC), triglyceride (TG), high density lipid-cholesterol (HDL-C), and low density lipid-cholesterol (LDL-C). Participants were classified as having diabetes if they had a FBG ≥ 7·0 mmol/L, were using diabetes medication, or had a history of diabetes [16]. Dyslipidemia was defined as having TC of ≥ 5·17 mmol/L, TG of ≥ 1·7 mmol/L, LDL-C of ≥ 3·37 mmol/L, HDL-C < 1·00 mmol/L or using dyslipidemia medication [17]. MetS was defined in accordance with the criteria of the American Heart Association scientific statements of 2009. Participants were considered to have MetS if they presented three or more of the following components: 1) elevated WC for Chinese individuals (≥ 85 cm in males; ≥ 80 cm in females), 2) elevated TG (≥ 1·7 mmol/L), or using lipid-lowering medication, 3) reduced HDL (< 1·0 mmol/L in males; < 1·3 mmol/L in females) or using lipid-lowering medication, 4) elevated BP (SBP ≥ 130 mm Hg and/or diastolic BP ≥ 85 mm Hg) or self-reported hypertension or taking antihypertensive medication, 5) elevated fasting glucose (≥ 5·56 mmol/L) or self-reported diabetes or using antidiabetic drugs. Family history of CVD refers to the self-reported CVD from at least one first-degree relative (biological parents, sibling) in the baseline survey.

Reference:

16. American Diabetes, A. Diagnosis and classification of diabetes mellitus. *Diabetes Care* **2014**, *37 Suppl 1*, S81-90, doi:10.2337/dc14-S081.

17. Expert Panel on Detection, E.; Treatment of High Blood Cholesterol in, A. Executive Summary of The Third Report of The National Cholesterol Education Program (NCEP) Expert Panel on Detection, Evaluation, And Treatment of High Blood Cholesterol In Adults (Adult Treatment Panel III). *JAMA* **2001**, *285*, 2486-2497, doi:10.1001/jama.285.19.2486.

Table S1. Association of the dietary pattern scores with CAS in the sensitivity analysis 1 (n= 13,989)

Dietary patterns	n	Increased cIMT			CP		
		Cases (%) (n = 3732)	Incident rate (events per 1000 person-years)	HR (95% CI)	Cases (%) (n = 2861)	Incident rate (events per 1000 person-years)	HR (95% CI)
**High-Fiber (range)** [a]							
Q1 (-3.44, -0.58)	3497	1387 (39.7)	133.6	Ref	1068 (30.5)	97.3	Ref
Q2 (-0.58, -0.32)	3496	670 (19.2)	63.1	0.48 (0.44, 0.53)	516 (14.8)	47.0	0.49 (0.44, 0.55)
Q3 (-0.32, 0.44)	3498	760 (21.7)	72.2	0.53 (0.48, 0.58)	579 (16.6)	53.1	0.53 (0.48, 0.59)
Q4 (0.44, 7.38)	3498	915 (26.2)	86.1	0.63 (0.57, 0.69)	698 (20.0)	63.1	0.63 (0.56, 0.70)
P for trend				< 0.0001			< 0.0001
**High-Fat (range)** [a]							
Q1 (-2.51, -0.69)	3498	525 (15.0)	48.7	Ref	417 (11.9)	37.8	Ref
Q2 (-0.69, -0.20)	3497	1137 (32.5)	106.7	2.24 (2.02, 2.49)	868 (24.8)	77.4	2.08 (1.85, 2.34)
Q3 (-0.20, 0.48)	3496	1087 (31.1)	105.7	2.22 (1.99, 2.47)	837 (23.9)	77.5	2.04 (1.81, 2.31)
Q4 (0.48, 13.1)	3498	983 (28.1)	94.2	2.02 (1.80, 2.27)	739 (21.1)	68.1	1.85 (1.62, 2.11)
P for trend				< 0.0001			< 0.0001
**High-Protein (range)** [a]							
Q1 (-3.29, -0.69)	3498	911 (26.0)	85.1	Ref	691 (19.8)	62.1	Ref
Q2 (-0.69, -0.10)	3497	904 (25.9)	86.5	1.01 (0.92, 1.11)	701 (20.1)	64.4	1.02 (0.91, 1.13)
Q3 (-0.10, 0.57)	3496	884 (25.3)	83.6	0.99 (0.90, 1.09)	689 (19.7)	64.0	1.01 (0.91, 1.13)
Q4 (0.57, 6.40)	3498	1033 (29.5)	99.1	1.10 (1.00, 1.22)	780 (22.3)	71.8	1.08 (0.96, 1.22)
P for trend				0.21			0.34
**High-Carbohydrate**							

**(range)** [a]							
Q1 (-3.32, -0.61)	3498	924 (24.8)	86.7	Ref	696 (19.9)	62.7	Ref
Q2 (-0.61, -0.05)	3497	895 (25.1)	85.3	1.05 (0.95, 1.15)	678 (19.4)	62.1	1.04 (0.94, 1.16)
Q3 (-0.05, 0.49)	3496	910 (26.4)	85.2	1.03 (0.94, 1.13)	694 (19.9)	62.6	1.03 (0.92, 1.14)
Q4 (0.49, 7.04)	3498	1003 (28.7)	97.2	1.17 (1.06, 1.28)	793 (22.7)	73.4	1.19 (1.07, 1.32)
P for trend				0.016			0.019
**DDS (range)** [b]							
Q1 [0.00, 1.00]	2925	986 (33.7)	110.1	Ref	754 (25.8)	80.6	Ref
Q2 [2.00]	7244	1743 (24.1)	78.9	0.74 (0.68, 0.80)	1,333 (18.4)	58.0	0.74 (0.68, 0.82)
Q3 [3.00]	3083	807 (26.2)	89.9	0.81 (0.73, 0.89)	624 (20.2)	66.9	0.83 (0.74, 0.93)
Q4 [4.00, 7.00]	737	196 (26.6)	92.5	0.77 (0.66, 0.90)	150 (20.4)	67.5	0.77 (0.64, 0.92)
P for trend				< 0.001			0.002
**DII (range)** [b]							
Q1 (-6.39, -2.26)	3560	930 (26.1)	86.7	Ref	701 (19.7)	62.9	Ref
Q2 (-2.26, -0.01)	3477	892 (25.7)	84.3	1.05 (0.95, 1.15)	688 (19.8)	62.6	1.07 (0.96, 1.19)
Q3 (-0.01, 2.22)	3490	872 (25.0)	83.7	1.16 (1.04, 1.28)	695 (19.9)	64.0	1.22 (1.09, 1.38)
Q4 (2.22, 6.37)	3462	1038 (30.0)	99.5	1.48 (1.32, 1.65)	777 (22.4)	71.3	1.45 (1.28, 1.65)
P for trend				< 0.0001			< 0.0001
**PURE (range)** [b]							
Q1 [0]	4431	1157 (26.1)	90.1	Ref	881 (19.9)	65.8	Ref
Q2 [1.0]	4902	1331 (27.2)	88.3	0.92 (0.85, 1.00)	1026 (20.9)	65.2	0.92 (0.84, 1.01)
Q3 [2.0]	2952	784 (26.6)	87.1	0.84(0.76, 0.93)	606 (20.5)	65.1	0.85 (0.76, 0.96)
Q4 [3.0, 5.0]	1704	460 (27.0)	88.0	0.83 (0.73, 0.94)	348 (20.4)	63.5	0.80 (0.70, 0.92)
P for trend				< 0.001			< 0.001

**hPDI (range)** [b]							
Q1 [36.0, 47.0]	3945	1208 (30.6)	101.2	Ref	923 (23.4)	74.0	Ref
Q2 (47.0, 49.0]	3408	874 (25.7)	85.5	0.88 (0.81, 0.96)	653 (19.2)	61.2	0.86 (0.78, 0.95)
Q3 (49.0, 51.0]	3273	842 (25.7)	87.3	0.90 (0.82, 0.98)	667 (20.4)	66.4	0.93 (0.84, 1.03)
Q4 (51.0, 64.0]	3363	808 (24.0)	78.1	0.81 (0.74, 0.88)	618 (18.4)	57.6	0.82 (0.74, 0.91)
P for trend				< 0.0001			0.001

Abbreviations: Q1 and Q4: the lowest and highest quartiles or groups of dietary pattern scores; BMI: body mass index; CAS: carotid atherosclerosis; CI: confidence interval; cIMT: carotid intima-media thickness; CP: carotid plaque; DDS dietary diversity score; DII: dietary inflammation index; hPDI: healthful plant-based diet index; HR: hazard ratio; PAR: population attributable risk; PURE: Prospective Urban Rural Epidemiology healthy diet.

a. Multivariable models are adjusted for age, sex, ethnicity, educational attainment, marital status, household income, physical activity, smoking status, drinking status, multivitamin supplement use, family history of cardiovascular disease, total energy intake, and each other dietary pattern scores (i.e., High-Fiber, High-Fat, High-Protein, and High-Carbohydrate).

b. Multivariable models are adjusted for age, sex, ethnicity, educational attainment, marital status, household income, physical activity, smoking status, drinking status, multivitamin supplement use, family history of cardiovascular disease, and total energy intake.

Table S2. Association of the dietary pattern scores with CAS in the sensitivity analysis 2 (n= 13,430)

Dietary patterns	n	Increased cIMT			CP		
		Cases (%) (n = 3173)	Incident rate (events per 1000 person-years)	HR (95% CI)	Cases (%) (n = 2861)	Incident rate (events per 1000 person-years)	HR (95% CI)
**High-Fiber (range)** [a]							
Q1 (-3.44, -0.58)	3358	1197 (37.7)	115.1	Ref	926 (38.3)	98.1	Ref
Q2 (-0.58, -0.32)	3357	557 (17.6)	53.5	0.49 (0.44, 0.54)	425 (17.6)	48.3	0.50 (0.44, 0.56)
Q3 (-0.32, 0.44)	3357	641 (20.2)	62.0	0.53 (0.48, 0.59)	482 (19.9)	54.3	0.53 (0.47, 0.59)
Q4 (0.44, 7.38)	3358	778 (24.5)	74.2	0.65 (0.58, 0.72)	587 (24.3)	64.4	0.64 (0.57, 0.72)
P for trend				< 0.0001			< 0.0001
**High-Fat (range)** [a]							
Q1 (-2.51, -0.70)	3357	425 (13.4)	40.4	Ref	334 (13.8)	38.8	Ref
Q2 (-0.70, -0.20)	3358	968 (30.5)	91.2	2.25 (2.00, 2.52)	748 (30.9)	78.6	2.15 (1.88, 2.45)
Q3 (-0.20, 0.48)	3358	936 (29.5)	91.7	2.34 (2.08, 2.64)	716 (29.6)	78.8	2.17 (1.89, 2.48)
Q4 (0.48, 13.1)	3357	844 (26.6)	81.9	2.10 (1.85, 2.39)	622 (25.7)	69.5	1.93 (1.67, 2.23)
P for trend				< 0.0001			< 0.0001
**High-Protein (range)** [a]							
Q1 (-3.29, -0.69)	3357	773 (24.4)	73.1	Ref	577 (23.8)	63.2	Ref
Q2 (-0.69, -0.10)	3358	771 (24.3)	74.8	1.03 (0.93, 1.14)	598 (24.7)	65.6	1.06 (0.95, 1.19)
Q3 (-0.10, 0.57)	3358	757 (23.9)	72.6	1.05 (0.94, 1.16)	584 (24.1)	64.2	1.09 (0.97, 1.23)
Q4 (0.57, 5.92)	3357	872 (27.5)	84.4	1.13 (1.02, 1.26)	661 (27.3)	72.8	1.15 (1.01, 1.30)

P for trend				0.051			0.056
**High-Carbohydrate (range)** [a]							
Q1 (-3.29, -0.61)	3358	796 (25.1)	75.9	Ref	602 (24.9)	64.3	Ref
Q2 (-0.61, -0.05)	3357	750 (23.6)	72.1	1.02 (0.92, 1.13)	562 (23.2)	63.1	1.00 (0.89, 1.13)
Q3 (-0.05, 0.49)	3357	771 (24.3)	73.1	1.03 (0.93, 1.15)	583 (24.1)	63.7	1.00 (0.90, 1.13)
Q4 (0.49, 7.04)	3358	856 (27.0)	83.8	1.16 (1.05, 1.29)	673 (27.8)	74.8	1.18 (1.05, 1.33)
P for trend				0.021			0.036
**DDS (range)** [b]							
Q1 [0.00, 1.00]	2793	854 (26.9)	96.7	Ref	656 (27.1)	82.4	Ref
Q2 [2.00]	6982	1481 (46.7)	67.8	0.75 (0.69, 0.82)	1124 (46.5)	59.0	0.75 (0.68, 0.83)
Q3 [3.00]	2952	676 (21.3)	76.3	0.82 (0.73, 0.91)	516 (21.3)	68.2	0.82 (0.73, 0.93)
Q4 [4.00, 7.00]	703	162 (5.11)	77.5	0.75 (0.63, 0.89)	124 (5.12)	69.2	0.74 (0.61, 0.91)
P for trend				< 0.001			0.002
**DII (range)** [b]							
Q1 (-6.39, -2.31)	3357	783 (24.7)	75.6	Ref	582 (24.1)	65.6	Ref
Q2 (-2.31, -0.05)	3358	756 (23.8)	71.7	1.00 (0.91, 1.11)	585 (24.2)	63.2	1.04 (0.93, 1.17)
Q3 (-0.05, 2.17)	3357	739 (23.3)	71.9	1.09 (0.97, 1.22)	580 (24.0)	65.3	1.13 (1.00, 1.29)
Q4 (2.17, 6.37)	3358	895 (28.2)	85.6	1.41 (1.25, 1.59)	673 (27.8)	71.8	1.40 (1.22, 1.61)
P for trend				< 0.0001			< 0.0001
**PURE (range)** [b]							
Q1 [0.00]	4240	966 (30.4)	76.2	Ref	881 (19.9)	67.4	Ref
Q2 [1.00]	4708	1,137 (35.8)	76.3	0.93 (0.85, 1.02)	1026 (20.9)	66.4	0.93 (0.84, 1.02)
Q3 [2.00]	2840	672 (21.2)	75.5	0.87 (0.78, 0.97)	606 (20.5)	66.3	0.88 (0.77, 0.99)

Q4 [3.00, 5.00]	1642	398 (12.5)	76.9	0.88 (0.77, 1.00)	348 (20.4)	64.6	0.84 (0.73, 0.98)
P for trend				0.018			0.015
**hPDI (range)** [b]							
Q1 [36.0, 47.0]	3763	1,026 (32.3)	87.1	Ref	735 (30.4)	75.8	Ref
Q2 (47.0, 49.0]	3288	754 (23.8)	74.6	0.87 (0.79, 0.96)	872 (36.0)	62.3	0.82 (0.74, 0.92)
Q3 (49.0, 51.0]	3140	709 (22.3)	74.4	0.89 (0.81, 0.98)	514 (21.2)	67.7	0.90 (0.81, 1.00)
Q4 (51.0, 64.0]	3239	684 (21.6)	66.8	0.79 (0.71, 0.87)	299 (12.4)	58.7	0.78 (0.70, 0.87)
P for trend				< 0.0001			< 0.001

Abbreviations: Q1 and Q4: the lowest and highest quartiles or groups of dietary pattern scores; BMI: body mass index; CAS: carotid atherosclerosis; CI: confidence interval; cIMT: carotid intima-media thickness; CP: carotid plaque; DDS dietary diversity score; DII: dietary inflammation index; hPDI: healthful plant-based diet index; HR: hazard ratio; PAR: population attributable risk; PURE: Prospective Urban Rural Epidemiology healthy diet.

a. Multivariable models are adjusted for age, sex, ethnicity, educational attainment, marital status, household income, physical activity, smoking status, drinking status, multivitamin supplement use, BMI, family history of cardiovascular disease, individual history of diabetes, hypertension, and dyslipidemia, total energy intake, and each other dietary pattern scores (i.e., High-Fiber, High-Fat, High-Protein, and High-Carbohydrate).

b. Multivariable models are adjusted for age, sex, ethnicity, educational attainment, marital status, household income, physical activity, smoking status, drinking status, multivitamin supplement use, BMI, family history of cardiovascular disease, individual history of diabetes, hypertension, and dyslipidemia and total energy intake.
